# Neural correlates of executive functions in patients with obesity

**DOI:** 10.7717/peerj.5002

**Published:** 2018-06-12

**Authors:** Ming-Chou Ho, Vincent Chin-Hung Chen, Seh-Huang Chao, Ching-Tzu Fang, Yi-Chun Liu, Jun-Cheng Weng

**Affiliations:** 1 Department of Psychology, Chung Shan Medical University, Taichung, Taiwan; 2 Clinical Psychological Room, Chung Shan Medical University Hospital, Taichung, Taiwan; 3 School of Medicine, Chang Gung University, Taoyuan, Taiwan; 4 Department of Psychiatry, Chang Gung Memorial Hospital, Chiayi, Taiwan; 5 Health Information and Epidemiology Laboratory, Chang Gung Memorial Hospital, Chiayi, Taiwan; 6 Center of Metabolic and Bariatric Surgery, Jen-Ai Hospital, Taichung, Taiwan; 7 Department of Medical Imaging and Radiological Sciences, Chung Shan Medical University, Taichung, Taiwan; 8 Department of Medical Imaging and Radiological Sciences, Chang Gung University, Taoyuan, Taiwan

**Keywords:** Diffusion tensor imaging, Obesity, Executive function, Resting-state functional magnetic resonance imaging, Generalized q-sampling imaging

## Abstract

Obesity is one of the most challenging problems in human health and is recognized as an important risk factor for many chronic diseases. It remains unclear how the neural systems (e.g., the mesolimbic “reward” and the prefrontal “control” neural systems) are correlated with patients’ executive function (EF), conceptualized as the integration of “cool” EF and “hot” EF. “Cool” EF refers to relatively abstract, non-affective operations such as inhibitory control and mental flexibility. “Hot” EF refers to motivationally significant affective operations such as affective decision-making. We tried to find the correlation between structural and functional neuroimaging indices and EF in obese patients. The study population comprised seventeen patients with obesity (seven males and 10 females, BMI = 37.99 ± 5.40, age = 31.82 ± 8.75 year-old) preparing to undergo bariatric surgery. We used noninvasive diffusion tensor imaging, generalized q-sampling imaging, and resting-state functional magnetic resonance imaging to examine the neural correlations between structural and functional neuroimaging indices and EF performances in patients with obesity. We reported that many brain areas are correlated to the patients’ EF performances. More interestingly, some correlations may implicate the possible associations of EF and the incentive motivational effects of food. The neural correlation between the left precuneus and middle occipital gyrus and inhibitory control may suggest that patients with a better ability to detect appetitive food may have worse inhibitory control. Also, the neural correlation between the superior frontal blade and affective decision-making may suggest that patients’ affective decision-making may be associated with the incentive motivational effects of food. Our results provide evidence suggesting neural correlates of EF in patients with obesity.

## Introduction

The prevalence of being overweight (BMI ≥ 25 kg/m^2^) and obesity (BMI ≥ 30 kg/m^2^) has more than doubled during the last three decades (WHO, 2016). Obesity is recognized as an important risk factor for many chronic diseases, such as cardiovascular disease, type 2 diabetes, hypertension, and cancer (Ko & Tang, 2007; Renehan et al., 2008; WHO, 2000).

Obesity and addiction have many shared neural circuits such as the mesolimbic “reward” neural system and the prefrontal “control” neural system (Chao et al., 2018; Gearhardt et al., 2011; Volkow et al., 2012, 2013; Volkow & Wise, 2005; Wang et al., 2004). Addiction is considered a disease of the brain’s reward system. For example, the functional connectivity strength of the putamen in the reward network (Kenny, 2011) was increased in the obese patients (García-García et al., 2013; Machann et al., 2013), whereas the caudate nucleus showed elevated task-related (viewing appetizing foods) functional connectivity with the amygdala and insula (Nummenmaa et al., 2012). Patients with obesity show atrophy in the frontal lobes, anterior cingulate cortex (ACC), hippocampus, and thalamus, in comparison to people with normal-weights (García-García et al., 2015; Raji et al., 2010). A recent review study (Carnell et al., 2012) suggests various impaired brain structures and functions in patients with obesity in reward (e.g., striatum, orbitofrontal cortex, and insula) and cognitive control and attention (e.g., PFC and ACC).

It remains unclear how the two neural systems correlate with the patients’ executive function (EF). This issue is critical, particularly if the mesolimbic reward neural system is related to the patients’ EF. This correlation suggests that the patients’ self-regulatory cognitive processes can be associated with the impulsive incentive motivational effects of food. We examined the correlations between the indices of structural and functional neuroimaging and EF performances in the patients with obesity.

Review papers (Fitzpatrick, Gilbert & Serpell, 2013; Smith et al., 2011) indicate that obese children, adolescents, and adults may have worse performance of EF. EF refers to a set of self-regulatory cognitive processes that are essential for adaptive behavior (Reinert, Po’e & Barkin, 2013; Pannacciulli et al., 2006). EF can be conceptualized as the integration of “cool” EF and “hot” EF (Noël, Brevers & Bechara, 2013; Zelazo et al., 2005). “Cool” EF is mediated by lateral inferior and dorsolateral frontostriatal and frontoparietal networks (Kerr & Zelazo, 2004), and refers to relatively abstract, non-affective operations (e.g., inhibitory control and mental flexibility) (Miyake et al., 2000). “Hot” EF is mediated by paralimbic orbitomedial and ventromedial frontolimbic structures, and refers to motivationally significant affective operations (e.g., affective decision-making) (Bechara et al., 2005; Damasio, Everitt & Bishop, 1996). We adopted the stop-signal task (Logan, Schachar & Tannock, 1997) and the color trails test (CTT) (D’Elia et al., 1996) to measure the inhibitory control of prepotent response and mental flexibility, respectively. We adopted the Iowa gambling task (IGT) (Bechara et al., 1994) to examine the affective decision-making ability.

Structural and functional brain imaging of patients with obesity and underlying their EF performances have not been sufficiently explored. The role of dopaminergic pathways in relation to the reward effects and control capability in obesity (Volkow, Wang & Baler, 2011; Volkow et al., 2012, 2013; Volkow & Wise, 2005) gave us great insights regarding the correlations between patients’ EF and their neural correlates. The lower-than-normal availability of dopamine D2 receptors in the striatum (reward system) of patients with obesity is associated with deteriorated activity in the PFC and the ACC (e.g., lower regional metabolic activity), resulting in deficient control capability over food overeating (Volkow et al., 2012, 2008). A negative correlation between the speed of mental processing and individual putamen (reward system) activation in patients with obesity has been observed (García-García et al., 2013).

In the current study, we used noninvasive diffusion tensor imaging (DTI), generalized q-sampling imaging (GQI), and resting-state functional magnetic resonance imaging (rs-fMRI) to find the correlation between structural and functional neuroimaging indices and EF in patients with obesity. The diffusion indices included fractional anisotropy (FA), mean diffusivity (MD), axial diffusivity (AD) and radial diffusivity (RD), generalized fractional anisotropy (GFA), normalized quantitative anisotropy (NQA), and isotropic value of the orientation distribution function (ISO). FA, GFA, and NQA are summary measures of microstructural integrity. While FA, GFA, and NQA are highly sensitive to microstructural changes, they are less specific to the type of change. MD and ISO are inverse measures of the membrane density; they are very similar for both gray and white matter and higher for cerebrospinal fluid (CSF). MD and ISO are sensitive to cellularity, edema, and necrosis. AD tends to be variable in white matter changes and pathology (e.g., AD decreases in axonal injury), and RD increases in white matter with de- or dys-myelination. Changes in the axonal diameters or density may also influence RD. The functional indices included amplitude of low frequency fluctuations (ALFF) and regional homogeneity (ReHo). ALFF is used to report the absolute intensity of spontaneous brain activity. The ALFF during resting state is considered to be physiologically meaningful, and reflective of spontaneous neural activity (Zhang et al., 2014). ReHo is based on the concept that blood oxygen level dependent signal fluctuations in a specific region reflect near neuronal activity arising at the same frequency, and that this temporal synchrony is confined to groups of neurons performing related functions (Philip et al., 2013). We correlated the above brain imaging indices with the EF (“cool” EF: inhibitory control and mental flexibility; “hot” EF: affective decision-making) in obese patients. We hypothesized that the activities of several brain areas in the mesolimbic reward (e.g., striatum, orbitofrontal cortex, and insula) and prefrontal control (e.g., PFC and ACC) systems are related to the patients’ EF performances.

## Materials and Methods

### Patients with obesity

A total of 17 right-handed patients with obesity (seven males and 10 females, BMI = 37.99 ± 5.40, age = 31.82 ± 8.75 year-old) aged between 23 and 54 years-old who were preparing to undergo bariatric surgery were recruited from an obesity clinic in a single regional teaching hospital. Informed consent was obtained from all patients, approved by the Institutional Review Board of Jen-Ai Hospital, Taichung, Taiwan (IRB No. 104-3465B). No patients took psychotropic agents during the study period and were not in need of immediate psychiatric intervention. Patients received structural, functional MRI, and EF tests before the bariatric surgery. The EF tests were performed before the actual scans were taken. Exclusion criteria of patients with obesity included: a history of another psychiatric disorder or substance dependence during the past year (except for dependence on caffeine or nicotine), any serious medical or neurological illnesses, current pregnancy or breastfeeding, and metallic implants or other contraindications to MRI. The exclusion criteria were confirmed by participants’ self-reports.

### The EF measures

The stop-signal task (Logan, Schachar & Tannock, 1997) is to measure the inhibitory control of prepotent behavior. Each trial began with a fixation for 250 ms, followed by a target shape. The primary task was to identify a square (length = 1.9°) or a circle (diameter = 2.1°). The instruction was to press the left mouse button to respond to a square and right mouse button to a circle. The patients were instructed to respond as accurately and quickly as possible. The target remained on the screen until there was a response, or until 1,250 ms had elapsed. Occasionally (25% of total trials) (Verbruggen, Logan & Stevens, 2008), a stop signal (75 ms) was presented shortly after the target onset in the primary task, when patients withheld their responses (a Stop trial). In the Stop trials, the temporal delay between the target onset and the stop signal onset (stop signal delay, SSD) was initially set at 250 ms. When response inhibition was successful, SSD increased by 50 ms; when response inhibition was unsuccessful, SSD decreased by 50 ms. There were 32 practice trials and three blocks of 64 formal trials (16 Stop trials and 48 Go trials). Between the blocks, patients had a 10-s break (Verbruggen, Logan & Stevens, 2008). The inter-trial interval was 2,000 ms. The dependent variable, the stop-signal reaction time (SSRT), was calculated by subtracting the SSD from RT. Higher SSRTs indicate longer time required to stop a prepotent response, thus indicating worse inhibitory control.

The CTT (D’Elia et al., 1996) is to measure mental flexibility. In CTT1, patients were given a page with scattered circles numbered from 1 to 25, with even-numbered circles colored yellow and odd-numbered ones colored pink. CTT1 required the individuals to connect numbers in ascending order from 1 to 25 as quickly as possible. The CTT2 also presented the patients with a page containing 25 circles, but on this sheet each color set was numbered. CTT2 required alternation between two different sets of stimuli. The task was to follow the number series with a pencil, but to alternate between the two colors as well (i.e., 1-pink, 2-yellow, 3-pink, and so on). The total time in seconds for CTT1and CTT2 was recorded, representing the CTT1 and CTT2 direct scores. The CTT score was calculated by subtracting CTT1 from CTT2 and then divided by CTT1 (i.e., (CTT2-CTT1)/CTT1). This CTT score was used to indicate the ability of mental flexibility; a higher score represented poorer flexibility.

The IGT (Bechara et al., 1994) is to examine affective decision-making ability. Over 100 trials, the patients had to make a choice between four decks of cards (A, B, C, and D), some of which yielded high immediate gain but larger future losses (“disadvantageous decks”: A and B), while others yielded lower immediate gain but smaller future losses (“advantageous decks”: C and D). The goal of the game was to win as much as possible. We used a global outcome score which was derived from the total number of cards chosen from the advantageous decks (C and D) minus the number of cards chosen from the disadvantageous decks (A and B) as a measure of the performance. A lower IGT score represented poorer affective decision-making ability.

### MRI data acquisition

All the images were acquired using a 1.5T MRI system (Ingenia, Phillips, Netherlands) with an 8-channel head coil. Echo planar diffusion images were obtained with TR/TE = 3,279/110 ms, resolution (voxel size) = 1.75 × 1.75 × 3 mm^3^, slices = 40, 67 non-collinear diffusion weighting gradient direction with *b* = 1,000 and 2,000 s/mm^2^ and one additional image without diffusion weighting (*b* = 0 s/mm^2^). The scan time of diffusion imaging for each patient was almost 21 min. Functional data were collected using echo-planar imaging sequences. All the patients were instructed to not focus their thoughts on anything in particular and to keep their eyes closed and remain awake during the resting state MR acquisition. For all the patients, the following sequence was used: TR/TE = 2,000/30 ms, resolution = 3.91 × 3.91 × 5 mm^3^, and the images were acquired in an interleaved order. Each brain volume comprised 20 axial slices and each functional run contained 400 image volumes, resulting in a scan time of 13.3 min for fMRI.

### Diffusion image analysis

Each patient’s original diffusion image was done via Eddy Current Correction using FSL (FMRIB Software Library, Oxford, UK). Then, the images were spatially normalized to the Montreal Neurological Institute (MNI) T2 weighted imaging (T2W) template using parameters determined from the normalization of the diffusion null image to the T2W template using statistical parametric mapping (SPM8, Wellcome Department of Cognitive Neurology, London, UK). The images were resampled with a final voxel size of 2 × 2 × 2 mm^3^. DTI and GQI reconstruction were performed using DSI Studio (National Taiwan University, Taipei, Taiwan). For the DTI analysis, the FA, MD, AD, and RD mapping were calculated. For the GQI analysis, the GFA, ISO, and NQA mapping were calculated.

### fMRI preprocessing

Preprocessing was carried out using a data processing assistant for resting-state fMRI (Chao-Gan & Yu-Feng, 2010) which is based on SPM8 and resting-state fMRI data analysis toolkit (REST, Lab of Cognitive Neuroscience and Learning, Beijing Normal University, China) (Song et al., 2011). After motion correction, functional images were coregistered to the T1 weighted structural image. The anatomical image was normalized to the MNI template using the diffeomorphic anatomical registration through exponentiated Lie algebra (Kong et al., 2014), and the resulting parameter file was used to normalize the functional images (voxel size = 3 × 3 × 3 mm^3^). Finally the normalized images were smoothed with a three-dimensional isotropic Gaussian kernel (full-width at half-maximum = 6 mm). Nuisance regression was performed using white matter, CSF, and the six head motion parameters as covariates. A temporal filter (0.01–0.12 Hz) was applied to reduce low frequency drifts and high frequency physiological noise.

### Amplitude of low-frequency fluctuations

We applied REST to calculate the ALFF. Briefly, the time courses were first converted to the frequency domain using a Fast Fourier Transform. The square root of the power spectrum was computed and then averaged across 0.01–0.12 Hz at each voxel; this averaged square root was taken as the ALFF. To reduce the global effects of variability across patients, the ALFF of each voxel was divided by the global mean ALFF for each patient, resulting in a relative ALFF. The relative ALFF in a given voxel reflects the degree of its raw ALFF relative to the average ALFF of the whole brain.

### Regional homogeneity

Regional homogeneity analysis was performed using the REST software. The linear trend of the time series was removed, and band-pass filtering (0.01–0.12 Hz) was performed to reduce the influence of physiological noise, such as the respiratory and cardiac rhythms. Individual ReHo maps were generated by assigning each voxel a value corresponding to Kendall’s coefficient of concordance (KCC) of its time series with its nearest 26 neighboring voxels. Then a mask was used to remove non-brain tissues and noise on the ReHo maps; for standardization purposes the individual ReHo maps were divided by their own mean KCC within the mask.

To evaluate the relationship between the brain structure, function and cognitive scores, multiple regression with false discovery rate correction was used to detect the correlation between DTI indices/GQI indices/ALFF/ReHo and SSRT/CTT score/IGT score for all the patients with SPM8. Age and gender were used as covariates. The multiple linear regression we used in SPM is the second level analysis, it is simply the entering of covariates (SSRT/CTT score/IGT score) which were tested for correlations with the signal change in contrasts across subjects (DTI indices/GQI indices/ALFF/ReHo). Age and gender were used as covariates regardless of the design that we choose.

## Results

### The EF measures

For the IGT and CTT we followed the literatures and the standards, there was not performance criteria for these two tasks. For the stop-signal task, all participants achieved 90% accuracy in the formal trials. For the IGT, CTT and the stop-signal task, we followed the literatures and the assessment standards, we did not discard faster or slower RTs. In the stop-signal task, the average SSRT was 247 ms, SD was 52.4 ms, and the range was between 153 and 378 ms. In the CCT, the average CCT score was 1.4, SD was 0.58, and the range was between 0.72 and 2.75. The average IGT score was 0, SD was 25.39, and the range was between −36 and 50.

### The correlation between the EF measures and the DTI indices

**Figure 1:**
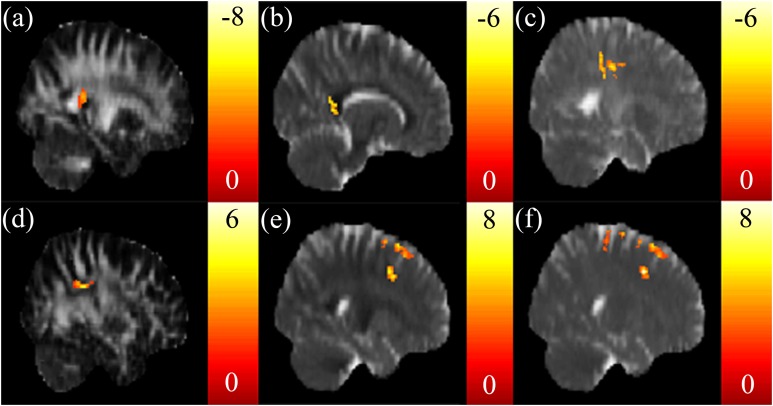
The correlation between the cognitive scores and the DTI indices. Significant negative correlations between stop signal reaction time (SSRT) and (A) FA in the right tapetum and (B) RD in the *corpus* callosum were found. (C) A significant negative correlation between CTT and AD in the left posterior corona radiata was found. Significant positive correlations between IGT and (D) FA in the right superior longitudinal fasciculus, (E) RD in the superior frontal blade, and (F) MD in the superior frontal blade were found. The color bar presents *t*-score. (*p* < 0.05, corrected).

A negative correlation between the SSRT and FA in the tapetum (Fig. 1A), and a negative correlation between the SSRT and RD in the *corpus* callosum (Fig. 1B) were reported. A negative correlation between the CTT score and AD in the posterior corona radiata was also found (Fig. 1C). There were no significant correlations between the CTT score and the FA, RD, and MD. A positive correlation was found between the IGT score and FA in the superior longitudinal fasciculus (Fig. 1D). A positive correlation between the IGT score and the RD, MD in the superior frontal blade (Figs. 1E and 1F) were obtained. There were no other significant correlations between the IGT score and the AD.

### The correlation between the EF measures and the GQI indices

**Figure 2:**
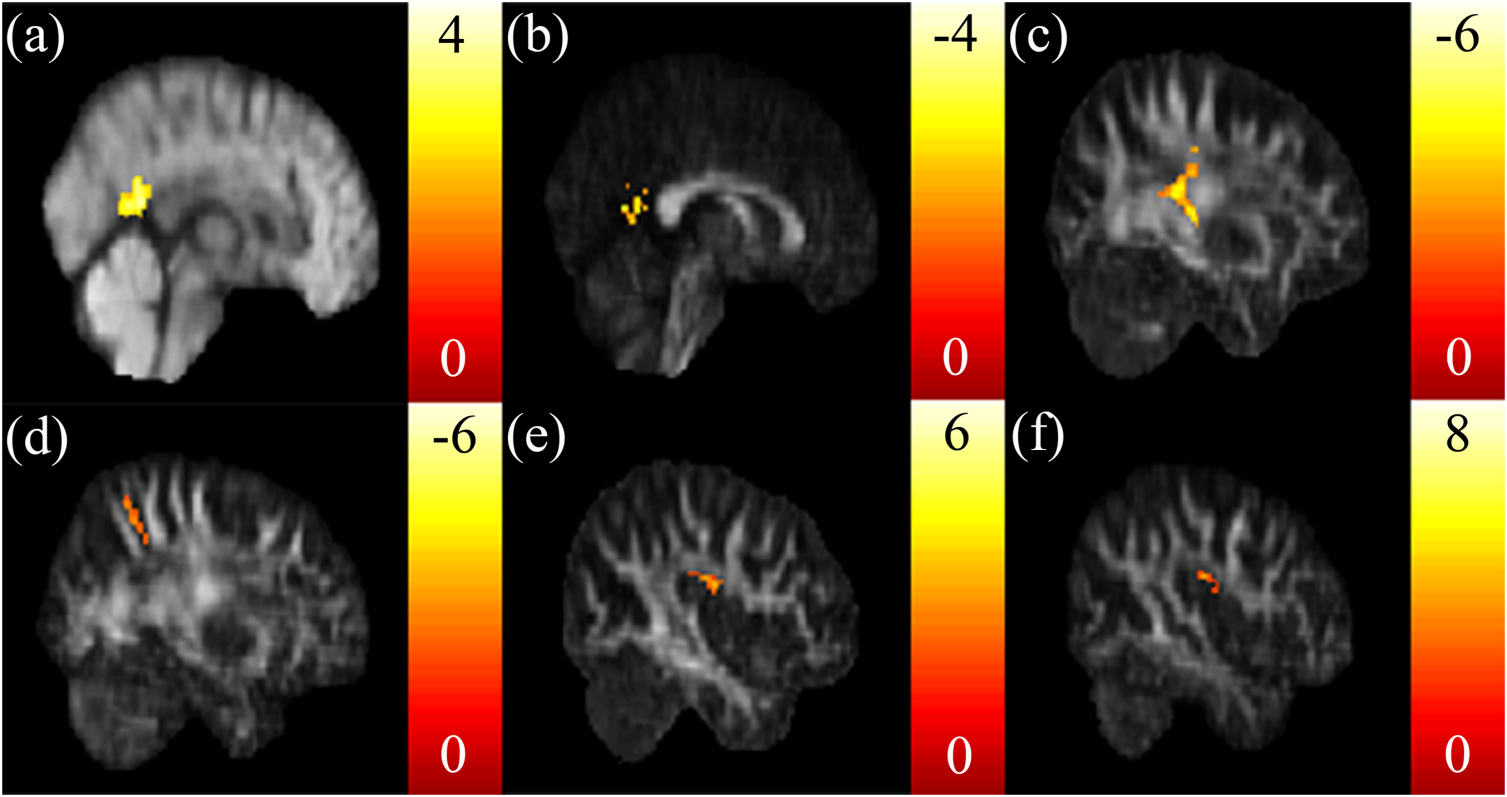
The correlation between the cognitive scores and the GQI indices. (A) Significant positive correlation between SSRT and ISO in the posterior cingulate, and (B) a significant negative correlation between SSRT and NQA in the posterior cingulate were found. Significant negative correlations between CTT and (C) GFA in the left posterior corona radiata and (D) NQA in the precuneus were found. Significant positive correlations between IGT and (E) GFA/(F) NQA in the right superior longitudinal fasciculus were found. The color bar presents *t*-score. (*p* < 0.05, corrected).

A positive correlation between the SSRT and ISO in the posterior cingulate was found (Fig. 2A). A negative correlation between the SSRT and NQA in the posterior cingulate was found (Fig. 2B). A negative correlation between the CTT score and GFA in the posterior corona radiata (Fig. 2C), and a negative correlation between the CTT score and NQA in the precuneus (Fig. 2D) were found. There were no other correlations between the CTT score and ISO. A positive correlation between the IGT score and GFA, NQA in the superior longitudinal fasciculus were found (Figs. 2E and 2F).

### The correlation between the EF measures and mfALFF

**Figure 3:**
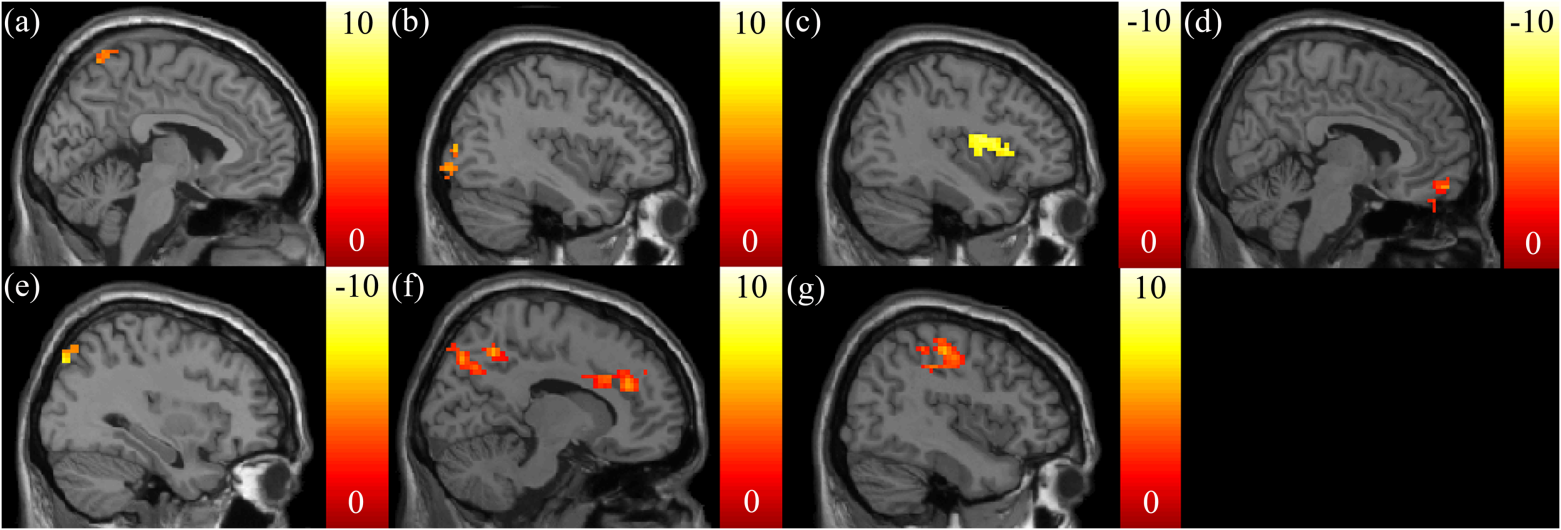
The correlation between the cognitive scores and the functional index, mfALFF. Highly positive correlations between the SSRT scores and mfALFF were found in the (A) left precuneus and (B) MidOG, whereas a highly negative correlation was observed in (C) insula. Negative correlations between the CTT scores and mfALFF in the (D) right vmPFC and (E) angular gyrus were found. Highly positive correlations between the IGT scores and mfALFF in the (F) ACC, right precuneus and (G) postcentral gyrus were found. The color bar presents *t*-score. (*p* < 0.05, corrected).

Positive correlations between the SSRT and mfALFF were found in the left precuneus (Fig. 3A) and middle occipital gyrus (MidOG) (Fig. 3B), whereas a negative correlation was observed in the insula (Fig. 3C). We found negative correlations only between the CTT score and mfALFF in the right vmPFC (Fig. 3D) and angular gyrus (Fig. 3E). We found positive correlations between the IGT score and mfALFF in the ACC, right precuneus (Fig. 3F) and postcentral gyrus (Fig. 3G).

### The correlation between the EF measures and mReHo

**Figure 4:**
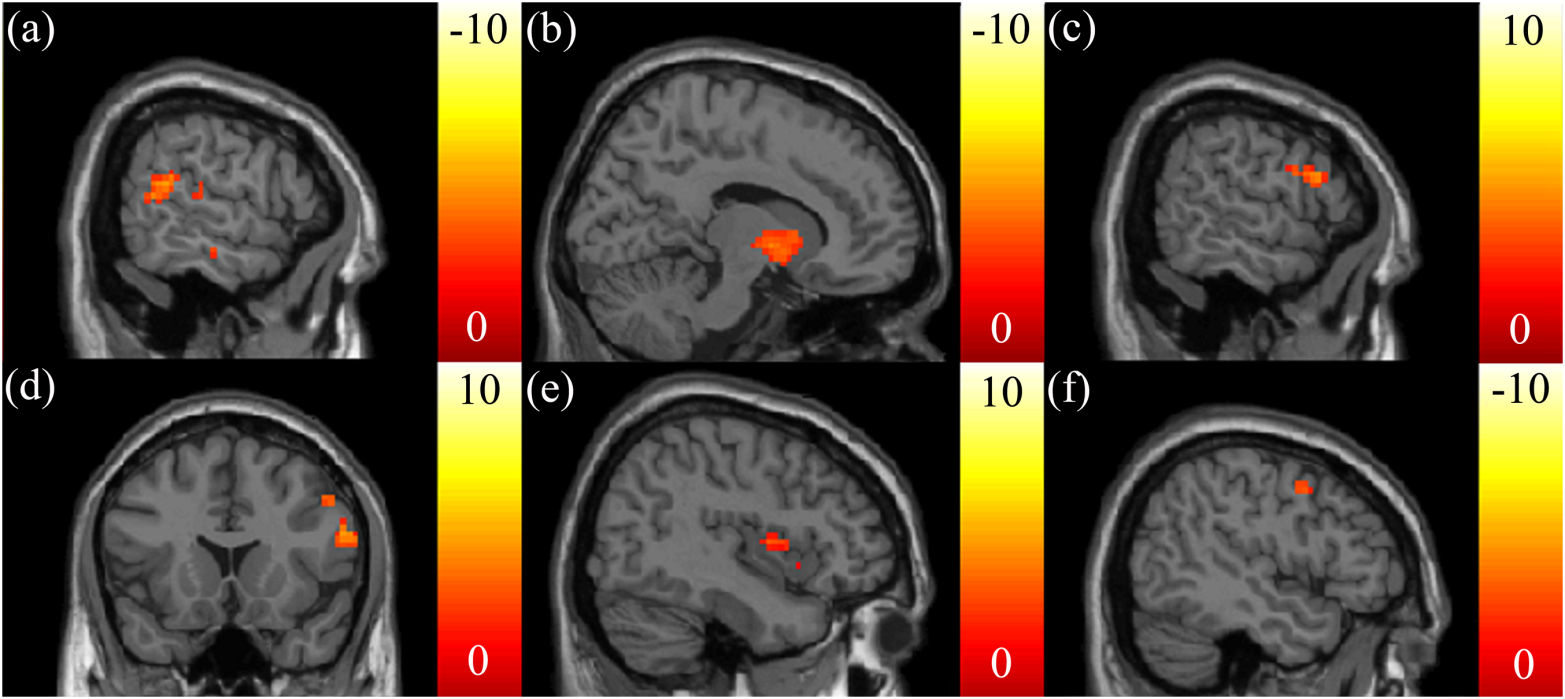
The correlation between the cognitive scores and the functional index, mReHo. Highly negative correlations between the SSRT scores and mReHo were found in the (A) right dmPFC and (B) putamen. (C, D) A highly positive correlation between the CTT and mReHo in the right OFC was found. (E) A highly positive correlation between the IGT scores and mReHo was found in the right insular, (F) whereas a highly negative correlation was found in the vmPFC. The color bar presents *t*-score. (*p* < 0.05, corrected).

Negative correlations between the SSRT and mReHo were found in the right dorsomedial prefrontal cortex (dmPFC) (Fig. 4A) and putamen (Fig. 4B). We also found a positive correlation between the CTT score and mReHo in the right OFC (Figs. 4C and 4D). A positive correlation between the IGT score and mReHo was found in the right insula (Fig. 4E), whereas a negative correlation was found in the vmPFC (Fig. 4F).

## Discussion

We correlated the indices of structural and functional brain imaging with the performances of “cool” and “hot” EF. Briefly, these correlations revealed that the patients’ EF can be associated with the impulsive incentive motivational effects of food.

### Inhibitory control and structural brain imaging

The SSRT was related negatively to the FA in the tapetum, and to the RD in the corpus callosum. The corpus callosum is a large bundle of commissural fibers that connects the left and right sides of the cerebral hemispheres, allowing for motor, sensory, and cognitive information between the brain hemispheres. The corpus callosum constitutes the tapetum (a white matter track) that projects from the splenium of the corpus callosum and connects both sides of the temporal lobe (Wakana et al., 2004). Worse inhibitory control (longer SSRT) is related to decreased diffusion anisotropy (lower FA). On the other hand, worse inhibitory control (longer SSRT) is related to better diffusion anisotropy (lower RD) in the corpus callosum.

The SSRT was related positively to ISO and negatively to NQA in the posterior cingulate cortex (PCC). The PCC is involved in voluntary control network (Leech & Sharp, 2014), mediating the “cool” EF (Kerr & Zelazo, 2004). Enhanced PCC activity facilitates stop-signal inhibition (Boehler et al., 2009; Hopfinger, Buonocore & Mangun, 2000). We also showed that the patients’ worse inhibitory control (longer SSRT) is related to decreased diffusion anisotropy (larger ISO and lower NQA) in the PCC.

### Inhibitory control and functional brain imaging

The SSRT was related positively to the mfALFF in the left precuneus and the MidOG, and negatively in the insula. The precuneus (Gearhardt et al., 2014; Tang et al., 2012) and the MidOG (Geliebter et al., 2006; Rothemund et al., 2007) are related to identifying the salience of visual, appetitive cues (e.g., food). The positive correlation suggests that the patients with obesity with better ability to detect appetitive food may have worse inhibitory control. This suggests that patients’ inhibitory control may be associated with the incentive motivational effects of food.

The insula plays a critical role in the generation of the conscious urge to use an addictive substance (Verdejo-Garcia, Clark & Dunn, 2012). Obese individuals showed increased activation in the insula (Volkow, Wang & Baler, 2011), making them favor food over other natural reinforcers. The insula is activated in response inhibition (Horn et al., 2003; Swick, Ashley & Turken, 2011). A recent study (Droutman, Read & Bechara, 2015) suggests that strong activation of the insula can be considered as increased engagement of the prefrontal control system. Patients with obesity with increased mfALFF in the insula had better inhibitory control (shorter SSRT).

The SSRT was related negatively to the mReHo in the right dmPFC and putamen. The dmPFC has been demonstrated to be critical in stop-signal response inhibition (Floden & Stuss, 2006; Venkatraman et al., 2009). Patients with obesity with increased mReHo in the dmPFC had better inhibitory control (shorter SSRT). The putamen involves in habitual drug- seeking and reinforcing learning (Koob & Volkow, 2010). Obese individuals showed enhanced functional connectivity strength in the putamen, may contribute to overeating (García-García et al., 2013). On the other hand, the basal ganglia circuitry (including the putamen) exerts an inhibitory influence on the presupplementary motor area and primary motor to mediate a response inhibition (Duann et al., 2009). Within the patients with obesity, increased mReHo in the putamen was related to better inhibitory control (shorter SSRT).

### Mental flexibility and structural brain imaging

The CTT score was related negatively to the AD in the posterior corona radiata, to the GFA in the posterior corona radiata, and to the NQA in the precuneus. The motor fibers are somatotopically arranged in the corona radiata (Kim & Pope, 2005). Overweight individuals (BMI = 25–30) had lower brain volumes in the corona radiata (Raji et al., 2010), which may disrupt their motor functions. Worse mental flexibility (larger CTT score) is related to possible disrupted motor functions, mediated by a decrease in diffusion anisotropy (lower AD and GFA) in the posterior corona radiata. The current study recruiting the patients with obesity and the study (Raji et al., 2010) recruiting the overweight individuals provide convergent evidence of possible disrupted brain area (e.g., posterior corona radiata) and motor functions.

The precuneus is related to identifying the salience of visual cues (Gearhardt et al., 2014; Tang et al., 2012), suggesting that it plays an important role visuospatial processing (e.g., directing attention in space and among objects) (Cavanna & Trimble, 2006). The CTT requires patients to deploy their attention to scan the circles with different numbers and colors (Rucklidge, 2006). Therefore, we suggest that worse mental flexibility (larger CTT score) is related to worse visuospatial processing, mediated by a decreased diffusion anisotropy in the precuneus.

### Mental flexibility and functional brain imaging

The CTT score was related negatively to the mfALFF in the right vmPFC and angular gyrus. Beside the critical role in affective decision-making (Koob & Volkow, 2010), patients with lesioned orbitalmedial PFC (the combination of the vmPFC and OFC territories) had difficulty acquiring simple rule change (e.g., the rule change in the CTT) (Zald & Andreotti, 2010). This negative correlation suggests that better capability to acquire rule change (larger mfALFF in the vmPFC) is related to better mental flexibility (smaller CTT scores).

The angular gyrus is involved in orienting spatiovisual attention to the relevant information (Seghier, 2013). A better ability to shift attention to the correct circles and numbers in the CTT (larger mfALFF of angular gyrus) is possibly related to better mental flexibility (smaller CTT scores).

The CTT score was related positively to the mReHo in the right OFC. The OFC has been implicated in many reward-based functions (Shott et al., 2015). The OFC involves mental flexibility: it enables the ability to shift away from previously learned responses (e.g., rule-shifting from CTT1 to CTT2) (Ragozzino, 2007). We found that increased mReHo of OFC was related to worse mental flexibility (longer CTT score), suggesting an inability to stop food intake (Rolls, 2008) and reduced reward sensitivity (Volkow et al., 2010). This result may reflect a possible deficit in patients’ OFC, leading to ineffective mental flexibility.

### Affective decision-making and structural brain imaging

The IGT score was related positively to the FA in the superior longitudinal fasciculus, and to the RD and MD in the superior frontal blade. The IGT score was also related positively to the GFA and NQA in the superior longitudinal fasciculus. The superior longitudinal fasciculus involves several functions, such as regulating motor behavior, visuospatial attention, working memory, and auditory information. An increase in the FA, GFA, and NQA in the superior longitudinal fasciculus is related to better affective decision-making (larger IGT score).

The obese individuals have been shown to have enhanced activations in the superior frontal blade when viewing pictures of high-calorie foods (Martin et al., 2010). The RD and MD are the inverse measurements of membrane density: larger values indicate lower myelination, dilute axonal packing, and larger extent of axonal degeneration. The positive correlation between the RD and MD in the superior frontal blade and the IGT score suggests that cue-evoked craving (food) may interfere with affective decision-making (lower IGT score). This suggests that patients’ affective decision-making (hot EF) may be associated with the incentive motivational effects of food.

### Affective decision-making and functional brain imaging

The IGT score was positively related to the mfALFF in the ACC, right precuneus and postcentral gyrus. The ACC is involved in inhibitory control/emotion regulation, conflict monitoring, compulsive behaviors, and impulsivity (Koob & Volkow, 2010; Noël, Brevers & Bechara, 2013; Volkow et al., 2012). The ACC is engaged in the IGT during the phase of anticipatory feeling and for implementing behavioral decisions (Li et al., 2010; Lin et al., 2008). The precuneus is activated after a risky choice during the IGT (Li et al., 2010; Lin et al., 2008). The postcentral gyrus is the location of the primary somatosensory cortex (S1). The sensory experience of palatable food plays a role in the development of obesity (DelParigi et al., 2005). This area may involve processing somatic signals for decision-making. Collectively, higher values of mfALFF in the ACC, right precuneus, and postcentral gyrus are related to higher IGT scores (better affective decision-making).

The IGT score was positively related to the mReHo in the right insula, but negatively related to the mReHo in the vmPFC. The insula plays a critical role in the generation of emotional/somatic states during the IGT, which is important for appropriate long-term decisions (Li et al., 2010). The vmPFC integrates the emotional/somatic states and working memory (e.g., online knowledge and information used during the deliberation of a decision) for decision-making (Li et al., 2010). The negative correlation between the mReHo in the vmPFC and the IGT score may result from possible impairment in this area in the patients with obesity. That is, in the patients with obesity, a higher value of mReHo in their impaired vmPFC may reflect the ineffective processing of affective decision-making. Future studies can examine this possibility in the patients with obesity.

### Limitations

Neural correlates in the current study are only exclusively for patients with obesity, normal weight or overweight individuals will be taken into consideration in the further study. The current fMRI results were mere associations between EF tests and functional activations of brain regions at rest. Causal inferences about ingestive behavior and reactions to food based on these associations between EF tests, that did not have food or related stimuli and rs-fMRI data cannot be made. Our study was limited in the small sample size of obese patients. It is difficult to recruit obese patients preparing for bariatric surgery given the smaller average BMI and obesity prevalence rate in Taiwan. This study was limited in lower external validity, since the participants were recruited only from the hospital. This limitation can be mitigated by recruiting participants from multiple sources. Since the brief experiment time, some cognitive variables that may relate to obesity such as intelligence and working memory capacity were not measured. A more comprehensive measures are suggested in the future. There was no pre-experiment instructions regarding caffeine or food consumption in the current study. It has been suggested that caffeine (Park et al., 2014) or glucose intake (Zanchi et al., 2018) is associated with cognitive performance and cortical activation.

## Conclusion

First, we found that many brain areas are correlated to the EF, consistent with previous studies. Secondly, in the patients with obesity, identifying the salience of appetitive cues (the precuneus and the MidOG) may be associated with “cool” EF (poor inhibitory control). In the patients with obesity, cue-evoked craving (the superior frontal blade) may be associated with "hot" EF (poor affective decision-making).

## Supplemental Information

10.7717/peerj.5002/supp-1 Supplemental Information 1 Table S1. The table summarizes the information of figures.

## Additional Information and Declarations

### Data Availability

The following information was supplied regarding data availability: Weng, JC (2018): obesity. figshare. Fileset. https://doi.org/10.6084/m9.figshare.6399965.v1.
