# Supplementary material for: Neural correlates of executive functions in patients with obesity
Source: PeerJ. 2018 Jun 12;6:e5002. doi: 10.7717/peerj.5002 (PMC6003388; doi:10.7717/peerj.5002)
Supplement: Supplemental Information 1 [file peerj-06-5002-s001.docx]

Table S1. The table summarizes the information of figures.

|  |  | \|T\| | \|Z\| | P_uncorr_ | P_corr_ |
| --- | --- | --- | --- | --- | --- |
| Fig. 1  (DTI) | (a) right tapetum | 3.98 | 3.12 | 0.001 | < 0.05 |
|  | (b) corpus callosum | 4.62 | 3.44 | 0.0001 | < 0.05 |
|  | (c) left posterior corona radiata | 4.81 | 3.53 | 0.0001 | < 0.05 |
|  | (d) right superior longitudinal fasciculus | 4.76 | 3.50 | 0.0001 | < 0.05 |
|  | (e) superior frontal blade | 6.62 | 4.22 | 0.0001 | < 0.05 |
|  | (f) superior frontal blade | 8.21 | 4.68 | 0.0001 | < 0.05 |
| Fig. 2  (GQI) | (a) posterior cingulate | 4.71 | 3.54 | 0.0001 | < 0.05 |
|  | (b) posterior cingulate | 4.06 | 3.20 | 0.001 | < 0.05 |
|  | (c) left posterior corona radiata | 3.92 | 3.23 | 0.001 | < 0.05 |
|  | (d) precuneus | 2.63 | 2.32 | 0.010 | < 0.05 |
|  | (e) right superior longitudinal fasciculus | 4.22 | 3.29 | 0.0001 | < 0.05 |
|  | (f) right superior longitudinal fasciculus | 4.52 | 3.44 | 0.0001 | < 0.05 |
| Fig. 3  (mfALFF) | (a) left precuneus | 4.83 | 3.54 | 0.0001 | < 0.05 |
|  | (b) MidOG | 4.78 | 3.51 | 0.0001 | < 0.05 |
|  | (c) insula | 8.17 | 4.66 | 0.0001 | < 0.05 |
|  | (d) right vmPFC | 4.07 | 3.21 | 0.001 | < 0.05 |
|  | (e) angular gyrus | 6.64 | 4.23 | 0.0001 | < 0.05 |
|  | (f) ACC and right precuneus | 4.63 | 3.45 | 0.0001 | < 0.05 |
|  | (g) postcentral gyrus | 4.21 | 3.28 | 0.0001 | < 0.05 |
| Fig. 4  (mReHo) | (a) right dmPFC | 4.82 | 3.62 | 0.0001 | < 0.05 |
|  | (b) putamen | 4.53 | 3.45 | 0.0001 | < 0.05 |
|  | (c) right OFC | 3.97 | 3.27 | 0.001 | < 0.05 |
|  | (d) right OFC | 4.79 | 3.51 | 0.0001 | < 0.05 |
|  | (e) right insular | 4.23 | 3.30 | 0.0001 | < 0.05 |
|  | (f) vmPFC | 4.03 | 3.16 | 0.001 | < 0.05 |
